# Supplementary material for: Insulin management in hospitalized patients with diabetes mellitus on high-dose glucocorticoids: Management of steroid-exacerbated hyperglycemia
Source: PLoS One. 2021 Sep 16;16(9):e0256682. doi: 10.1371/journal.pone.0256682 (PMC8445406; doi:10.1371/journal.pone.0256682)
Supplement: S1 File — (PDF) [file pone.0256682.s001.pdf]

General Medicine/Surgical Floor Analog Insulin Inpatient Glucose Control Guidelines at Cook County Health System

1. Order Blood Glucose (BG) fingersticks TID AC & bedtime if eating meals or order BG fingersticks Q 6 hrs if nutrition per tube feedings, TPN, IV with dextrose or NPO.
2. Order Baseline Hemoglobin A1c
3. Start insulin on any patient with a random BG > 200mg/dL or a pre-prandial BG > 180 mg/dL x 2 readings within 24 hours. Use insulin both basal (on **all** patients) and prandial (only with meals). Choose supplemental insulin algorithm (order the same insulin as prandial) according to daily insulin requirements (see below).
4. Targets are **BG 140-180mg/dL** pre-prandial and fasting periods. Stable patients with low risk of hypoglycemia can be kept in the 110-140 mg/dL range.
5. Reassess patients **every 24 hours** and incorporate patient's supplemental requirements into scheduled insulin by adding the total supplemental doses received over 24 hours, divide the total by 3 (i.e. 3 meals) and add each to the prandial doses

Diabetes Analog Insulin Protocol For Type 1 and Type II

**Initiating Insulin**	▪ Total daily dose (TDD) can be calculated based on weight:<br>**for GFR>30ml/min** calculate **0.5 units/kg/day**,**for GFR<30ml/min** calculate**0.25 units/kg/day**. One half of TDD should be basal insulin, remaining half is prandial divided by 3 into TID AC<br>▪ Or if patient had been on insulin at home: Discuss and verify the home dose with the patient. After verifying dose with patient add up the total daily dose and order: 50% as basal insulin (Glargine) and 50% as prandial (Lispro) divided by three (TID AC) if eating. Hold prandial insulin if NPO.<br>▪ Oral meds: discontinue metformin; may continue short acting glipizide if criteria not met for insulin. If criteria for initiating insulin are met, start insulin, discontinue glipizide
**Basal**	▪ **Begin Glargine (Lantus): Routine dose at 9:00 p.m.**<br>**Type 2 DM**: Initial dose according to time of order placement.<br>If order placed between:<br>❖ 12 noon until 9 p.m. give scheduled full dose at 9 p.m.<br>❖ 9 p.m. until 2 a.m. give full dose of Glargine ordered as **NOW**<br>❖ 2 a.m. until 12 noon give 1/3 of dose ordered as **NOW** then the **full dose** that evening at 9 p.m.<br>**(1/3 of the dose is given once for immediate basal requirements)**<br>**Type 1 DM**: Give the full dose regardless of the time of patient arrival
**Prandial & Supplemental (patient eating)**	▪ **Begin Lispro (Humalog) TID AC Prandial and Supplemental:**<br>If pre-prandial BG is not at goal, add PRN supplemental Lispro to prandial dose (see supplemental algorithms on page 3).<br>If initiating insulin while NPO then follow guidelines below (page 2) for prolonged NPO.
**Precautions:**	➢ **Dose reduction of 50% is also recommended for: 1) hepatic impairment 2) hypoglycemia prone patients (i.e,weight based insulin calculated at 0.25 units/kg/day).**

	➢ **If TPN or tube feedings stopped or patient is made NPO after insulin given start D5W**<br>➢ **If treatment with steroid is tapered or discontinued patient will require less insulin, doses may need aggressive reduction or insulin discontinued if not required previously**

**NPO & Prolonged NPO**	▪ <u>CONTINUE BASAL INSULIN</u><br>▪ <u>PRANDIAL LISPRO INSULIN (TID AC)</u> is held during NPO or for a clear liquid diet (Clear Liquid Diet has insufficient calories to be considered a meal and is treated the same as NPO)<br><br>▪ <u>SUPPLEMENTAL LISPRO INSULIN</u>: is used during prolonged NPO (miss 3 meals or more). When ordering supplemental in place of prandial insulin, the order should be every 6 hours PRN for BG>200mg/dL, begin with Low Dose Algorithm, order accuchecks every 6 hours. Recommend ordering an IV with dextrose

<u>NPO, Tube Feeds, TPN</u>

**Tube Feedings**<br><br>**Or**<br><br>**TPN**	▪ If continuous tube feedings or TPN is initiated and blood glucose results satisfy criteria for insulin, use initial basal dosing for Glargine and use supplemental Lispro:<br>1. Order Low Dose Lispro supplemental algorithm in PRN orders to be given every 6 hours for BG >200mg/dL<br>2. Order accuchecks to be done every 6 hours<br>3. Upward titrate basal insulin (Glargine) for blood glucose control by calculating the total supplemental Lispro required for 24 hours and adding 50% of that total to Glargine.<br><br>▪ If tube feedings are given per bolus then continue TID AC guidelines for Glargine and Lispro.<br><br>▪ If tube feedings or TPN are interrupted or discontinued then decrease insulin dose and/or:<br>1. If discontinuing tube feedings then begin IV with dextrose (D5)<br>2. If discontinuing TPN then begin IV with dextrose (D10) at the same rate that TPN had been given.

Supplemental Algorithm for Hyperglycemia (Lispro/Humalog) – give ***in addition*** to prandial insulin

Pre-meal BG (mg/dL)	**Very Low Dose Algorithm** <15 units insulin/day	**Low Dose Algorithm** 15-40 units insulin/day	**Medium Dose Algorithm** 40-80 units of insulin/day	**High Dose Algorithm** >80 units of insulin/day
150-199	0 unit	1 unit	1 unit	2 unit
200-249	1 unit	2 units	3 units	4 units
250-299	1 unit	3 units	5 units	6 units
300-349	2 units	4 units	7 units	8 units
>349	2 units (>400 call MD)	5 units	9 units	10 units

Insulin Adjustment Based on BG

Abnormal Blood Glucose	Insulin Dose to Adjust	Meal to Target
Pre-lunch	Pre-breakfast RA* insulin	Breakfast or mid- morning snack
Pre-dinner	Pre-lunch RA* insulin	Lunch +/- mid afternoon snack
Bedtime	Pre-dinner RA* insulin	Dinner
Morning Fasting BG	Evening Glargine	Bedtime snack

*RA = RAPID ACTING

Adjusting Basal Insulin

Blood glucose target	Glargine insulin dose adjustment
If FBG is <50 mg/dL or severe hypoglycemia event	Decrease dose by 50%
If FBG is < 50 to 70 mg/dL	Decrease dose by 20%
If FBG is 70-100mg/dL	Decrease by 10%
If FBG is >180mg/dL and < 200 mg/dL and no hypoglycemic episodes	Increase dose by 10% of the previous dose
If FBG is > 200 mg/dL and <250 mg/dL and no hypoglycemic episodes	Increase dose by 20% of the previous dose
If FBG is > 250 mg/dL and no hypoglycemic episodes	Increase dose by 30% of the previous dose

Adjusting Prandial Insulin

Prandial insulin is adjusted according to the amount of supplemental insulin given the day before. If the pre-prandial BG is > than 150mg/dL and the patient was given supplemental doses of insulin the day before, the total of all supplemental doses from the day before will be divided by 3 and added to each prandial dose of insulin.

If no supplemental dose was given the day before and the non-fasting pre-prandial blood glucose is above 150mg/dL, the prandial dose may be increased by 10% of the previous dose. Please ensure supplemental is given.
