# Supplementary material for: Insulin management in hospitalized patients with diabetes mellitus on high-dose glucocorticoids: Management of steroid-exacerbated hyperglycemia
Source: PLoS One. 2021 Sep 16;16(9):e0256682. doi: 10.1371/journal.pone.0256682 (PMC8445406; doi:10.1371/journal.pone.0256682)
Supplement: Attachment — Submitted filename: Response to Reviewers 7-28-21.docx [file pone.0256682.s003.docx]

PONE-D-21-07298R1
Insulin management in hospitalized patients with diabetes mellitus on high-dose glucocorticoids: Management of steroid-exacerbated hyperglycemia
PLOS ONE

Review Comments to the Author

Please use the space provided to explain your answers to the questions above. You may also include additional comments for the author, including concerns about dual publication, research ethics, or publication ethics. (Please upload your review as an attachment if it exceeds 20,000 characters)

Reviewer #1: The authors have addressed my comments.

I would consider adding a reference to an ongoing randomized clinical trial to address The Management of Glucocorticoid-Induced Hyperglycemia in Hospitalized Patients: <https://clinicaltrials.gov/ct2/show/NCT01810952>

***This ongoing trial is now referenced in the Discussion section.***

I would also add that there is some data to suggest that the insulin dosing pattern should be tailored to match the glucocortoid dosing and type (e.g. optimal dosing for prednisone may be different than for dexamethasone). Hence, a single protocol for all inpatients on steroids may not be optimal.
<https://eje.bioscientifica.com/view/journals/eje/179/4/EJE-18-0315.xml>

***We’ve added reference to this approach in our Introduction.***

Reviewer #2: The authors have not yet sufficiently addressed the comment regarding the decision to convert glucocorticoid doses to prednisone equivalent within the text of the manuscript. The rationale provided in the response to reviewers should be included within the methods section.

***The rationale provided in our prior response is now included in the Methods section.***
